# Incidence and Comorbidity of Dementia with Lewy Bodies: A Population-Based Cohort Study

**DOI:** 10.1155/2018/7631951

**Published:** 2018-05-29

**Authors:** Sheng-Kung Yang, Weishan Chen, Chun-Hsien Su, Chung-Hsiang Liu

**Affiliations:** ^1^Department of Neurology, Chang Bing Show Chwan Memorial Hospital, Changhua County, Taiwan; ^2^Management Office for Health Data, China Medical University Hospital, Taichung, Taiwan; ^3^College of Medicine, China Medical University, Taichung, Taiwan; ^4^Department of Exercise and Health Promotion, College of Education, Chinese Culture University, Taipei, Taiwan; ^5^Department of Neurology, China Medical University Hospital, Taichung, Taiwan

## Abstract

**Background and Aims:**

Dementia with Lewy bodies (DLB) is the third most common form of dementia. Epidemiological studies of DLB in Taiwan are scarce. In this study, we estimated the incidence of DLB and comorbidity in the population of Taiwan.

**Methods:**

Data were obtained from the Taiwan National Health Insurance Research Database (NHIRD). DLB patients between 2000 and 2013 were enrolled in assessments of incidence and comorbidity.

**Results:**

The incidence of DLB was shown to be 7.10 per 100,000 person-years (95% CI = 6.63–7.59), which increased with age. The average age at diagnosis was 76.3, and this was higher for males than for females. The comorbidity rates of hypertension and hyperlipidemia in DLB patients were higher in females than in males.

**Conclusions:**

Epidemiologic data from large-scale retrospective studies is crucial to the prevention of DLB.

## 1. Introduction

Dementia is a major health issue around the globe. Affected individuals can have considerable difficulty in their daily functioning, due to an inability to maintain cogent thought processes or keep their memories in order [1]. Dementia with Lewy bodies (DLB) is the third most common form of dementia after Alzheimer's disease and vascular dementia [2]. In America, DLB has been shown to influence 10–25% of cases of dementia, affecting 1%-2% of the population aged over 65 years [3]. The comedian Robin Williams suffered from DLB at the time of his suicide in 2014 [4]. Lewy bodies are abnormal microscopic deposits that consist primarily of alpha-synuclein (a protein found widely in the brain). They were discovered by Frederick H. Lewy, M.D., in the early 1900s [5]. These abnormal aggregations lead to the gradual destruction of certain brain cells over time, resulting in a progressive decline in reasoning capacity and the ability to function independently.

The fact that DLB is a degenerative dementia with alpha-synuclein pathology makes it clinically different from other types of dementia [6]. The distinctive clinical features include visual hallucinations, Parkinsonism, cognitive impairment with fluctuations, autonomic dysfunction, rapid eye movement sleep behavior disorder (RBD), and neuroleptic sensitivity. Parkinson's disease with dementia (PDD) is another major neurocognitive disorder with Lewy bodies. DLB and PDD share many clinical and neuropathological features. It is always a challenge for physicians to differentiate DLB from PDD. Clinically, patients with DLB present with less tremor but more orthostatic hypotension, delusion, attention fluctuation, and visual hallucination. Poor balance and early onset of dementia are more frequent in DLB. However, patients with DLB vary in the severity of these symptoms, and many individuals lack standard signs in the early stages of the disease. Furthermore, because of the morphological heterogeneity of the DLB/PDD group within synucleinopathies, it would be difficult to differentiate DLB from PDD simply from neuropathological findings without sufficient clinical data [7]. This means that no single test or combination of tests can conclusively diagnose DLB in a living patient. As a result, DLB is often misdiagnosed or progresses unrecognized, according to the criteria last revised in 2015 [2, 8, 9]. Palmqvist et al. found that more than 50% of cases with DLB go undiagnosed [10]. This led to the establishment of new criteria at the 2017 International Dementia with Lewy Bodies (DLB) Conference [11]. The new criteria feature sensitivity of 83% and specificity of 95%, based on research confirmed by postmortem autopsy [12]. So far, there is no clear or objective distinction between the two entities established, except the arbitrary timing of the appearance of cognitive impairments and Parkinsonism (1-year rule) [13].

One population-based prospective cohort study estimated the incidence of suspected DLB at 112/100,000 person-years among individuals aged 65 and over [14]. A systemic review revealed that DLB incidence ranges from 50 to 160 per 100,000 person-years [15]. Currently, very few studies focused on comorbidities of DLB. There was one linkage study indicating a worse comorbidity profile in DLB patients, with a higher prevalence of depression, stroke, and migraine, compared with the AD population [16]. Awareness of DLB has increased following the adoption of revised diagnostic criteria and the publishing of new studies reporting primary data; however, epidemiological and comorbidity studies of DLB in Taiwan remain scarce. In this study, we investigated the incidence and comorbidity of DLB in a national retrospective study.

## 2. Data Source

The Taiwanese government established the National Health Insurance (NHI) in 1995, and more than 99% of the population of Taiwan is currently enrolled [17]. Research institutes under the NHI have released databases to promote research as subsets of the National Health Insurance Research Database (NHIRD). The Longitudinal Health Insurance Database 2000 (LHID2000) includes a random selection of 1 million patients from the NHIRD. From this database, we were able to obtain registration and claim information of patients. To ensure the privacy of patients, personal ID numbers were reencoded prior to the release of information. This study was approved by the International Review Board, China Medical University and Hospital Research Ethics Committee (IRB permit number: CMUH-104-REC2-115).

## 3. Sampled Participant, Relevant Variables, and Comorbidities

In this study, DLB was defined as having been diagnosed based on ICD-9-CM code 331.82 or following the 1-year rule or having been diagnosed with Parkinson's disease (PD) or Parkinsonism (ICD-9-CM codes 332 and 333, excluding 333.1–333.8) within 1 year before or after the development of dementia (ICD-9-CM codes 290.0, 290.1, 290.2, 290.3, 294.1, and 331.0) [11]. The index date was the date of diagnosis according to ICD-9-CM code 331.82 or diagnosis with dementia. Patients who had been diagnosed with stroke (430–435), head injury (850–854, 959.01), or hydrocephalus (331.3, 331.4, 331.5, 741.0, and 742.3) prior index date were excluded.

The comorbidities discussed for DLB patients were diabetes mellitus (DM) (ICD-9-CM code 250), hypertension (HTN) (ICD-9-CM codes 401–405), hyperlipidemia (ICD-9-CM code 272), coronary artery disease (CAD) (ICD-9-CM codes 410–414), congestive heart failure (CHF) (ICD-9-CM code 428), and chronic kidney disease (CKD) (ICD-9-CM code 585). According to the location of insurance coverage, the patients were classified within Northern, Central, Southern, or Eastern Taiwan.

## 4. Statistical Analysis

We began by calculating the incidence of DLB between 2000 and 2013 according to the number of patients diagnosed with DLB during that period divided by the number of individuals in the LHID2000 in each year of the study period. We assembled 95% confidence intervals of incidence according to Poisson distribution. We compared the demographics of male and female DLB patients using the chi-squared test for categorical variables and the *t*-test for continuous variables. Data analysis in this study was performed using SAS statistical software (Version 9.4 for Windows; SAS Institute Inc., Cary, NC, USA), with statistical significance set at a *p* value < 0.05.

## 5. Results

Between 2000 and 2013, 872 patients were newly diagnosed with DLB, which translated to an incidence rate of 7.10 per 100,000 person-years (95% CI = 6.63–7.59). The incidence rates among males and females were 7.06 and 7.14, respectively, per 100,000 person-years. The incidence rate increased with age regardless of sex. The incidence was higher in Southern and Eastern Taiwan than in other areas.

**Figure 1:**
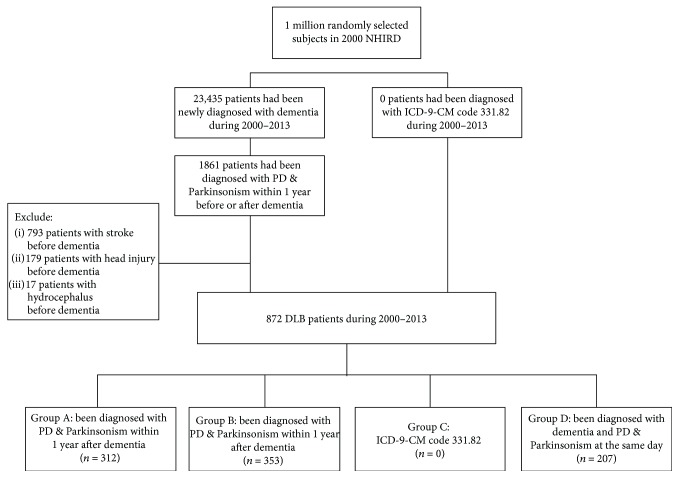
Flowchart of DLB patient selection from the National Health Insurance Research Database in Taiwan.

Then patients were divided into four groups according to the type of DLB with which they had been diagnosed as shown in Figure 1. Patients in Group A were diagnosed with PD within 1 year following diagnosis with dementia, whereas those in Group B were diagnosed with Parkinsonism within 1 year prior to the onset of dementia. Patients in Group C were diagnosed with DLB according to ICD-9-CM code 331.82. Patients in Group D were diagnosed with dementia and PD and/or Parkinsonism on the same day. We identified no patients diagnosed with DLB using ICD-9-CM code 331.82. Most of the patients belonged to Group A or B, regardless of gender, age, area, or the level of hospital. We also identified many patients in Group D. As shown in Table 1, the mean age of patients diagnosed with DLB was 76.3, and the average age was higher for males than for females. The mean age at the time of death was 83.3. Among patients with a history of HTN, 74.9% were diagnosed with DLB. We observed no significant differences between male and female patients with regard to the comorbidities DM, CAD, CHF, or CKD. A higher proportion of female patients presented HTN or hyperlipidemia.

## 6. Discussion

DLB is the second most common cause of degenerative dementia; however, there has been a dearth of research into the incidence of DLB in Taiwan. Rongve and Aarsland found its incidence at 0.7–1.4 new cases/100,000 person-years [18]. Another geographically defined population in Olmsted County, Minnesota (MN), reported an overall DLB incidence of 3.5 per 100,000 person-years, which increased sharply with age [19].  Table 2 lists the age-, sex-, and area-specific incidence rates (new cases per 100,000 person-years) of DLB in Taiwan. Between 2000 and 2013, the overall incidence of DLB in Taiwan was 7.10 per 100,000 person-years (95% CI = 6.63–7.59), and the incidence was slightly lower among men than among women (7.06 versus 7.14, resp.). The incidence of DLB increased with age, ranging from 0.81 among those <65 years old and peaking at 122 among those >85 years old, with an overall DLB incidence of 244.5 cases per 100,000 person-years among subjects aged 65 and older. The overall incidence of DLB was shown to increase consistently with age. In Southern Taiwan, the overall incidence of DLB was 9.62 per 100,000 person-years (95% CI = 8.56–10.8), which was higher than in other regions of Taiwan. The incidence values in this study may have been influenced by ascertainment bias. Rather than adopting a clinical or pathological diagnosis of DLB, we defined DLB based on ICD-9 and diagnoses of Parkinsonism as well as dementia within 1 year. It is therefore possible that cases of early onset DLB without Parkinsonism or dementia may have been missed. It is also possible that we failed to exclude other non-DLB diseases presenting both Parkinsonism and dementia. Thus, our estimates pertaining to the incidence of DLB must be regarded as imprecise. Nonetheless, these are currently the only large-population estimates in Taiwan. One previous study found that among patients with DLB, the median age at the time of death was 78 years [20]. In the present study, the mean age at death was 83.3 years (Table 2).

DLB is difficult to differentiate from Alzheimer's disease (AD) or PD with dementia due to similarities in cognitive and motor symptoms.  Table 3 shows that no patients were diagnosed with DLB using ICD-9-CM code 331.82 in Taiwan. This cannot be attributed to an inability to diagnose patients with DLB. Other explanations are at work. First, ICD-9-CM codes 294.20 (dementia without behavioral disturbance) and 294.21 (dementia with behavioral disturbance) were widely used as an alternative to definite diagnostic codes. Second, cholinesterase inhibitors and memantine are the only medications currently available for patients with dementia. Unfortunately, those medications are not covered by the NHI, even after DLB is clinically diagnosed. It is for this reason that ICD-9-CM code 331.82 is not used for DLB in Taiwan. This left us no alternative but to select Groups A, B, and D in order to form a cohort of DLB patients for subsequent investigations.

HTN is a major risk factor for cerebrovascular disorders; however, it increases the risk of cognitive decline and dementia. A growing body of evidence indicates that chronic HTN is an important factor associated with the microstructural damage of brain white matter and cognitive decline [21]. Control of HTN is a key intervention in the prevention of dementia, and the effects are similar to those obtained from most antihypertensive agents [22, 23]. Nonetheless, a very limited number of studies have investigated the association between HTN and DLB. One nationwide, population-based, cross-sectional study of dementia among individuals aged 65 years and over in Taiwan revealed that 52.94% of patients with dementia presented HTN levels exceeding those in the population with normal cognition [24]. As shown in Table 1, there is a high rate of HTN as a comorbidity of DLB (74.9%), and the proportion of HTN is higher among males versus among females (71.5% versus 78.3%, resp.). These results imply that the proportion of HTN in patients with DLB is higher among females However, there is little evidence concerning the association between DLB and NTN and the underlying mechanisms.

Lewy bodies composed of alpha-synuclein fibrils are abundant in the cortical neurons of patients with DLB. Evidence suggests that the interaction between alpha-synuclein and lipids on the cell membrane can trigger alpha-synuclein aggregation [25]. However, the relationship between hyperlipidemia and cognition remains an issue of controversy. One large study of older adults identified a correlation between higher low-density lipoproteins and better cognitive performance [26]. A longitudinal study of 1159 elderly Chinese people found that higher amounts of low-density lipoproteins are associated with accelerated cognitive decline [27]. However, a meta-analysis of four large randomized trials among patients diagnosed with AD revealed that lipid-lowering therapy using statins had no effect on cognition in the treatment or prevention of dementia [28]. One study of dementia among a population aged 65 years and over in Taiwan revealed that the proportions of patients with hyperlipidemia comorbidity are similar among those with dementia and among those with normal cognition (16.79% and 18.71%, resp.). In this study, we found that 39.0% of DLB patients also presented with hyperlipidemia and that the rates were higher among females. Interestingly, no previous studies have focused on the association between hyperlipidemia and DLB.

This study was subject to a number of limitations that warrant consideration. First, diagnosing DLB is difficult, and the definition of DLB in this study inevitably leads to the identification of non-DLB patients and patients in the early stages of DLB. Second, we were unable to obtain information on factors that could affect the results, such as duration of the comorbidity and use of medication.

This is the first large-population retrospective study on the incidence of DLB and cardiovascular comorbidities in Taiwan. Our results showed the incidence of DLB and higher comorbidity HTN and hyperlipidemia in female DLB patients in Taiwan. In the future, more generalizable studies concerning the association between DLB and vascular risk factors and cardiovascular disease are warranted.

## Figures and Tables

**Table 1:** Characteristics of DLB.

	Total	Male	Female	*p* value
	(*n* = 872)	(*n* = 438)	(*n* = 434)	
Age				
<65	89 (10.2)	40 (9.13)	49 (11.3)	0.27
65–74	246 (28.2)	117 (26.7)	129 (29.7)	
75–84	397 (45.5)	202 (46.1)	195 (44.9)	
≥85	140 (16.1)	79 (18.0)	61 (14.1)	
Mean (SD)	76.3 (9.84)	77.1 (9.48)	75.6 (10.1)	0.03
Death	176 (20.2)	95 (21.7)	81 (18.7)	0.27
Mean age at death (SD)	83.3 (6.56)	83.5 (6.24)	83.1 (6.95)	0.70
Comorbidity				
Diabetes mellitus	230 (26.4)	104 (23.7)	126 (29.0)	0.08
Hypertension	653 (74.9)	313 (71.5)	340 (78.3)	0.02
Hyperlipidemia	340 (39.0)	148 (33.8)	192 (44.2)	0.002
CAD	420 (48.2)	201 (45.9)	219 (50.5)	0.18
CHF	148 (17.0)	66 (15.1)	82 (18.9)	0.13
CKD	70 (8.03)	40 (9.13)	30 (6.91)	0.23

CAD: coronary artery disease; CHF: congestive heart failure; CKD: chronic kidney disease.

**Table 2:** Incidence rate of DLB.

	2000–2013			
	*n*	Cases	Incidence per 100,000 person-years	(95% CI)
Total	12,284,820	872	7.10	(6.63–7.59)
Sex				
Male	6,202,935	438	7.06	(6.42–7.75)
Female	6,081,806	434	7.14	(6.48–7.84)
Age				
<65	10,968,984	89	0.81	(0.65–1.00)
65–74	760,233	246	32.4	(28.4–36.7)
75–84	440,724	397	90.1	(81.4–99.4)
≥85	114,879	140	122	(103–144)
Area				
Northern	5,602,310	309	5.52	(4.92–6.17)
Central	2,531,483	169	6.68	(5.71–7.76)
Southern	3,140,566	302	9.62	(8.56–10.8)
Eastern & island	1,010,198	92	9.11	(7.34–11.2)

**Table 3:** Classified DLB patients.

	2000–2013				
	Cases	Group			
		A	B	C	D
		*n* (%)	*n* (%)	*n* (%)	*n* (%)
Total	872	312 (35.8)	353 (40.5)	0 (0)	207 (23.7)
Sex					
Male	438	155 (35.4)	179 (40.9)	0 (0)	104 (23.7)
Female	434	157 (36.2)	174 (40.1)	0 (0)	103 (23.7)
Age					
<65	89	41 (46.1)	26 (29.2)	0 (0)	22 (24.7)
65–74	246	98 (39.8)	97 (39.4)	0 (0)	51 (20.7)
75–84	397	132 (33.2)	167 (42.1)	0 (0)	98 (24.7)
≥85	140	41 (29.3)	63 (45.0)	0 (0)	36 (25.7)
Area					
Northern	309	112 (36.2)	112 (36.2)	0 (0)	85 (27.5)
Central	169	51 (30.2)	80 (47.3)	0 (0)	38 (22.5)
Southern	302	115 (38.1)	123 (40.7)	0 (0)	64 (21.2)
Eastern & island	92	34 (37.0)	38 (41.3)	0 (0)	20 (21.7)
Accreditation level of hospital					
Medical center	217	77 (35.5)	83 (38.2)	0 (0)	57 (26.3)
District hospital	343	116 (33.8)	139 (40.5)	0 (0)	88 (25.7)
Local hospital	197	69 (35.0)	87 (44.2)	0 (0)	41 (20.8)
Other	114	50 (43.9)	44 (38.6)	0 (0)	20 (17.5)

Group A: been diagnosed with PD & Parkinsonism within 1 year after dementia. Group B: been diagnosed of PD & Parkinsonism within 1 year before dementia. Group C: ICD-9-CM code 331.82. Group D: been diagnosed with dementia, PD, & Parkinsonism at the same day

## Data Availability

The data used to support the findings of this study are available from the corresponding author upon request.
